# Emotional Face Recognition in Children With Attention Deficit/Hyperactivity Disorder: Evidence From Event Related Gamma Oscillation

**DOI:** 10.18869/nirp.bcn.8.5.419

**Published:** 2017

**Authors:** Mahdiyeh Sarraf Razavi, Mehdi Tehranidoost, Farnaz Ghassemi, Parivash Purabassi, Athena Taymourtash

**Affiliations:** 1. Department of Neurosciences and Addiction Studies, School of Advanced Technologies in Medicine, Tehran University of Medical Sciences, Tehran, Iran.; 2. Department of Psychiatry, School of Medicine, Tehran University of Medical Sciences, Tehran, Iran.; 3. Research Center for Cognitive and Behavioral Sciences, Tehran University of Medical Sciences, Tehran, Iran.; 4. Department of Biomedical Engineering, Amirkabir University of Technology, Tehran, Iran.

**Keywords:** Emotional face recognition, Event-Related Oscillation (ERO), Gamma band activity, Attention Deficit Hyperactivity Disorder (ADHD)

## Abstract

**Introduction:**

Children with attention-deficit/hyperactivity disorder (ADHD) have some impairment in emotional relationship which can be due to problems in emotional processing. The present study investigated neural correlates of early stages of emotional face processing in this group compared with typically developing children using the Gamma Band Activity (GBA).

**Methods:**

A total of 19 children diagnosed with ADHD (Combined type) based on DSM-IV classification were compared with 19 typically developing children matched on age, gender, and IQ. The participants performed an emotional face recognition while their brain activities were recorded using an event-related oscillation procedure.

**Results:**

The results indicated that ADHD children compared to normal group showed a significant reduction in the gamma band activity, which is thought to reflect early perceptual emotion discrimination for happy and angry emotions (P<0.05).

**Conclusion:**

The present study supports the notion that individuals with ADHD have some impairments in early stage of emotion processing which can cause their misinterpretation of emotional faces.

## 1. Introduction

A DHD is a common neurodevelopmental disorder characterized by inattentiveness and hyperactivity/impulsivity (American Psychiatric Association, 2013). Individuals with ADHD also show problems in social and emotional functions, including the effective assessment of the emotional state of others. It is important to set the adaptive behavior of human facial expressions in social interactions (Cadesky, Mota, & Schachar, 2000; Corbett & Glidden, 2000). Based on the evidence, frontotemporal-posterior and fronto striatal cerebellar systems are involved in emotional functions. These regions may contribute to impairments of emotional recognition in ADHD (Corbett & Glidden, 2000; Dickstein, Bannon, Xavier Castellanos, & Milham, 2006; Durston, Van Belle, & De Zeeuw, 2011).

However, neural studies to investigate these impairments are rare. Results of recent studies revealed that gamma band activity (GBA) in electroencephalography (EEG) as well as Event-Related Potentials (ERP) play an important role in evaluation of higher cognitive processes such as attention, memory, language, and emotion (Fell et al., 2001; Keil et al., 2001; Sebastiani, Simoni, Gemignani, Ghelarducci, & Santarcange-lo, 2005; Matsumoto, Ichikawa, Kanayama, Ohira, & Iidaka, 2006; Martini et al., 2012). Several studies report that the gamma band activity at 40 Hz oscillation is a correlate of selective attention (Lutzenberger, Pulvermüller, Elbert, & Birbaumer, 1995; Keil et al., 2001; Keil, Stolarova, Moratti, & Ray, 2007; Martini et al., 2012).

Also results of studies indicate that an increase in gamma band activity at 40 Hz frequency is associated with the tuning of novelty processing by negative emotions (Singer, 1995; Garcia-Garcia, Yordanova, Kolev, Domínguez-Borràs, & Escera, 2010). Interestingly, results of Janik, Rezlescu, & Banissy (2015) study indicates that modulating occipital gamma with 40 Hz using transcranial Alternating Current Stimulation (tACS) enhances facial anger perception. Balconi and Pozoli (2009) showed an increased gamma band activity in response to emotions (happiness, sadness, fear, and anger) compared with neutral stimuli. Keil et al., (2007) found an increase in synchrony of very early cortical oscillations at 20–35 Hz aversive visual stimuli.

Müller, Gruber, & Keil (2000) showed an increased gamma band activity (30±50 Hz) for negative valence over the left temporal region as compared to the right one and a lateral shift towards the right hemisphere for positive valence. Martini et al., (2012) found that unpleasant images compared to neutral visual stimuli, elicited an increase of gamma power (30–45 Hz) at 200 ms and 850 ms after stimulus presentation.

Time course and specific topography of affective gamma band activity modulations during cognitive stimuli demonstrate discrimination between early and late processing stages. Basar (2012) reported possibility of 3–4 phase/time-locked gamma responses in 28–45 Hz frequency window during presentation of stimuli. According to their explanation, the early response, starts at 100 ms, in the primary occipital cortex which is probably the direct response over the short pathway via lateral geniculate nucleus and reflects the early visual processing.

According to Keil et al., (2007), the early gamma response could be sensory in origin and sensitive to simple features associated with emotions. Balconi and Pozilli (2009) reported an increasing early gamma band response for emotional stimuli between 150 ms and 250 ms. On the other hand, according to Basar (2012), late processing stage, starting around 300 ms, can be associated with the conscious perception and discrimination of the emotional stimuli. According to Polich (2007) study, time window 300–500 ms onset stimuli is modulated by allocation of attention, initial memory storage, and processing of cognitive tasks.

Martini et al., (2012) found two peaks of weak activation at high gamma frequencies (65–80 Hz) between 200 and 400 ms after stimulus onset; it is interpreted to be less sensitive to image features and more dependent on the conceptual processing of the stimulus identity. Balconi and Lucchiari (2008) reported higher GBA for emotional and neutral stimuli and this increase was more pronounced between 250 and 350 ms. And finally, time window around 400 to 800 ms reflect more strategic high-level processes that require the conscious awareness such as decision making, response criterion, and deep and elaborate processing of emotion (Schupp et al., 2004; Williams et al., 2007). Accordingly, these findings show an important role of gamma oscillations in evaluating early and late stages of cognitive stimuli.

On the other hand, abnormalities in gamma band response and phase synchronization have been shown to be related to various neurological and psychiatric disorders such as schizophrenia, Alzheimer, and ADHD (Matsumoto et al., 2006; Basar & Guntekin, 2013). Gross et al., (2012) reported reduced gamma oscillations in emotional face perception in Autism Spectrum Disorder. Studies show that ADHD patients have lower absolute and/or relative gamma power in comparison with age-matched healthy controls (Cited in Basar & Guntekin, 2013). Few studies indicate impaired gamma band responses related to cognitive stimuli in ADHD patients compared to healthy controls (Yordanova, Banaschewski, Kolev, Woerner, & Rothenberger, 2001; Lenz et al., 2008; Lenz et al., 2010). Lenz et al., (2010) reported ADHD group (11–17 years old) showed no differentiation between known and unknown stimuli during application of forced-choice–reaction task. However, normal group revealed increased evoked gamma response following familiar stimuli compared to new images.

Based on the mentioned studies, gamma band activity could reflect the characteristics of emotional integration or emotional utilization processes in individuals with ADHD. To our knowledge and according to review articles by Basar and Guntekin (2013) and Güntekin and Başar (2014), our study is the first one that evaluates gamma-band responses with stages of neural activity of emotional face recognition in children with ADHD. We expected that gamma band activity would be diminished for emotional faces in these patients compared to typically developing children during stages of facial emotional recognition. The present study aimed to gain better understanding of the neurobiological basis of children with ADHD in emotional face processing.

## 2. Methods

### 2.1. Participants

Nineteen boys, aged between 7 and 11 years (Mean±SD age: 9.21(1.13) years) diagnosed with ADHD were compared with 19 typically developing ones (9.73±1.04 years) matched with age, sex, and years of education. The control group was recruited from elementary schools in Tehran. Children with ADHD were recruited from patients referred to a child and adolescent psychiatric clinic. The individuals with ADHD were diagnosed according to DSM-IV (Statistical Manual of Mental Disorders, Fourth edition) (American Psychiatric Association, 2013). The patients were diagnosed as combined type and were drug naive.

Conners’ Parent Rating Scale-Revised (CPRS-R, short version) was administered to the participants to confirm the diagnosis of ADHD and determine severity of their symptoms. If the T-scores of CPRS-R subscales were above 65, the participant was excluded from the normal group. The two groups were right-handed, had corrected to normal visual acuity. Intelligence Quotient (IQ) of all participants were evaluated according to the WISCR IQ test (ADHD group: 106±4.36, Control group: 122±10.71) (Table 1).

**Table 1.** Clinical and demographic characteristics.

	**Diagnosis**				**F**	**P**

	**TD (n=19)**		**ADHD (n=19)**			

	**Mean**	**SD**	**Mean**	**SD**		
Age, y	9.68	1.05	9.15	1.16	0.1	0.7
Full-scale IQ	122	10.7	106	4.73	10	0.003
Conners’-oppositional	47	6.7	77	6.1	1.38	0.2
Conners’ -inattentive	47	6.2	75	10.4	3.75	0.03
Conners’ -hyperactive	48	8.8	76	4.7	2.003	0.04
Conners’ -ADHD index	45	5.6	78	7.8	4.23	0.04

Conner’s: Conners’ parent rating scale revised.

### 2.2. Task and stimuli

A compilation of 6 Caucasian faces (3 females and 3 males) in JPG format expressing happy, angry, sad, and neutral expressions were collected from Cohn Kanade AU-coded Facial Expressions Database (Kanade, Cohn, & Tian, 2000). Luminance and contrast of all images were equivalent across stimuli using Photoshop (version 7). The photos were in black and white and positioned within a rectangular frame (261×365 pixel array). The pictures were presented for 2000 ms at the center of the monitor screen and instantly replaced by a white fixation point in the light gray background (1024×768 pixels). The inter stimulus interval (ISI) was 1400±100 ms. The task was designed using Eevoke software (version 3.1).

The main task included 1 practice and 5 experimental blocks. Each experimental block comprised with 48 trials; 4 emotions (anger, happiness, sadness, and neutral) of each faces (3 female and 3 males) that repeated two times. Thus, there were 60 repeats of each expression in a random way. We defined four buttons for each facial expressions procedure (anger, happiness, sadness, and neutral) on a joy stick.

All participants were invited to the laboratory of EEG recording. The parents completed a consent form before starting the examination. During the EEG session, participants were seated in a comfortable chair in a dimly lit room 60 cm from a 17-inch LG computer screen. The participants were asked to look at the center of the screen during the recordings, if possible without making eye movements, and to blink only during the intervals. To ensure that they attend to stimuli, they were monitored by camera in another control room during task performance. All children were instructed to press on the button for each expression when they recognized the target stimuli during each trial (Stimuli presentation until the end of fixation).

### 2.3. Electrophysiological recording and analysis

Continuous EEG signals were recorded by 64 Ag/ AgCl electrodes mounted in an electrode cap (Waveguard, ANT, Netherlands) according to the international 10–20 standard and additional intermediate positions in neuropsychology laboratory of Payam-e Noor University. ASA 4.7.1 software was used for data acquisition. Electrode impedances were maintained below 10 kΩ. The sampling rate was 512 Hz. A 50-Hz notch filter of the recording system, eliminated the line noise during signal acquisition. Event-related oscillations data were analyzed offline using MATLAB R2013a software.

Raw data were filtered with a band-pass filter of 0.1 to 80 Hz and referenced to the mastoids average. The eye movement artifacts were canceled using the independent component analysis. In addition, the remaining artifacts with deflection amplitudes of ±100 μV from the baseline were eliminated (primarily through automatic artifact reduction). Artifact-free EEG recordings were then segmented into epochs ranging from 200 ms prestimulus to 800 ms poststimulus. Each channel baseline epoch was corrected by prestimulus average voltage subtraction. Several studies reported that the lower frequencies of Gamma band oscillation (about 40 Hz), modulated with emotional stimuli indicated attentional processing and discrimination of the emotional stimuli (Keil et al., 2001; Keil et al., 2007; Martini et al., 2012; Herrmann, Munk, & Engel, 2004).

To investigate facial emotion processing based on gamma oscillations, we constructed a new time series by concatenating a specific time window from all clean epochs. Then we estimated the signal power spectrum in frequency band of 35–45 HZ for three different time windows based on literature (Martini et al., 2012) and following current data analysis: 0–250 ms as early processing (can be sensitive to simple features associated with emotions (Keil et al., 2007)), 250–500 ms as late processing (can indicate allocation of attention, initial memory storage the processing of cognitive tasks (Polich, 2007)), and 500–750 ms as later ones (can reflect more strategic high-level processes that require the conscious awareness and deeper and elaborate processing of emotion (Schupp et al., 2004; Williams et al., 2007)). Based on the previous studies, F3, F4, C3, C4, P3, P4, O1, O2 electrodes were used for the statistical analysis (Ramos-Loyo, González-Garrido, Sánchez-Loyo, Medina, & Basar-Eroglu, 2009).

### 2.4. Statistical analysis

Frequency-band measures were statistically analyzed using repeated-measure analysis of variance (ANOVA) with the following core factors: facial expression (happiness, anger, sadness, and neutral), site (area) (anterior [F3, F4], central [C3, C4], occipital [O1, O2], and parietal [P3, P4]), side (lateralization) (left [F3, C3, P3, O1], and right [F4, C4, P4, O2]) as the within subjects factors, and groups (patients and controls) as the between subject factor. Greenhouse Geisser correction was used for the degrees of freedom. In the next step, paired t-test were used to break down between main effects such as site and independent sample t test were used to break down between-subject and interaction effects such as facial expression×group. Throughout the experiment, P<0.05 were considered significant.

## 3. Results

We carried out repeated-measure ANOVA (facial expression×site×side×group [4×4×2×2]) for three time windows separately. In the first time window (0–250 ms), this analyses revealed a significant main effect of group (F_1, 36_=12.36, P=0.001; partial η2=0.155), and a significant main effect of site (F_2.1, 76.26_=3.78, P=0.02; partial η2=0.013). Also the results showed a significant interaction effect of site×group (F_2.1, 76.26_=3.79, P=0.02; partial η2=0.059), and a significant interaction effect of facial expression×group (F_2.1, 77.47_=4.68, P=0.01; partial η^2^=0.069). In the second (250–500 ms) and third (500–750 ms) time windows, repeated-measure ANOVA revealed only main effect of group which showed smaller gamma band activity in ADHD group compared to healthy control one (P<0.05). We observed no significant main effect or interaction effect in terms of other factors of side, site, face expression, and group (P>0.05) in the second and third time windows. Therefore, our results focused on first time window that indicated early facial emotion processing.

**Figure 1.**
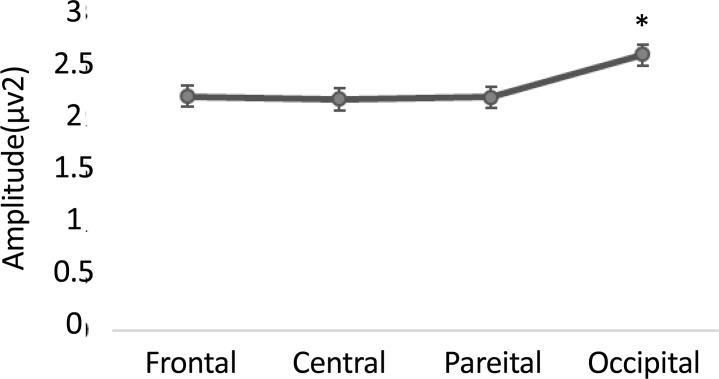
Mean (standard error) of gamma band activity in different areas in response to facial expressions.

**Figure 2.**
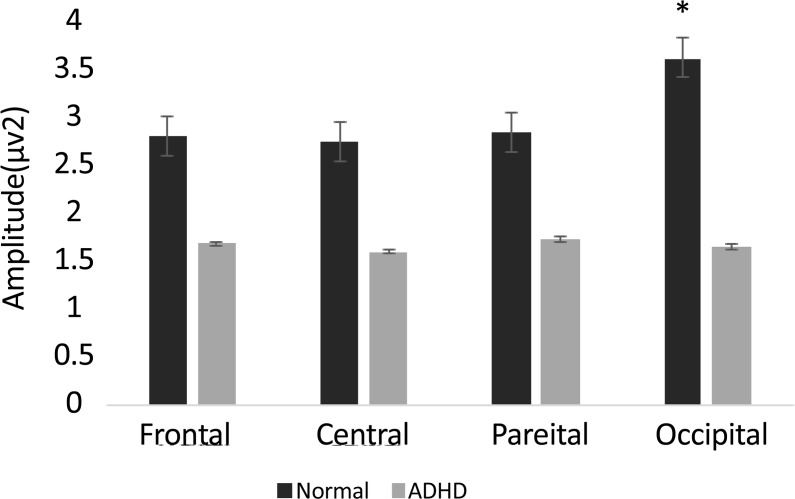
Mean (standard error) of the gamma band activity in different sites in response to facial expressions.

**Figure 3.**
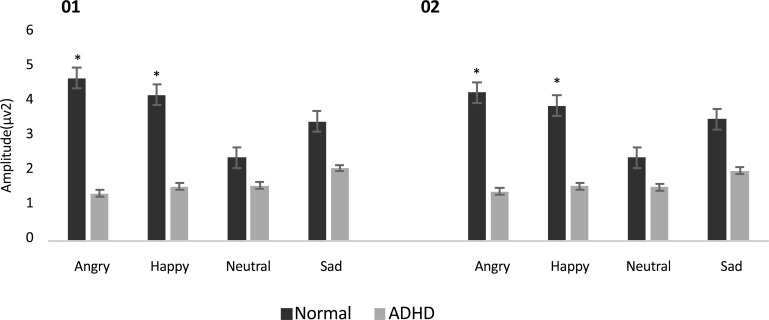
Mean (standard error) of the gamma band activity in left and right hemispheres (O1, O2) in response to facial expressions among the ADHD and control groups.

Also post hoc analysis showed that gamma oscillation measures were smaller in ADHD group (1.6±0.2) compared to normal group (2.9±0.2) in the first time window. Follow-up paired t test showed greater gamma band activity in occipital site compared to frontal (P=0.05, t(37)=1.97), central (P=0.01, t(37)=2.47), and parietal (P=0.02, t(37)=2.28) areas in ADHD group (Figure 1). Follow-up independent t test revealed a significant lower gamma band activity in ADHD group compared to normal children in occipital regions (F_4.2, 36_=14.8, P=0.001) (Figure 2). Also follow-up independent t test showed a significant decrease for happiness (F_2.6, 36_=6.63, P=0.01), (F_3.2, 36_=6.8, P=0.01) and anger (F_4.2, 36_=13.7, P=0.001), (F_6, 36_=8.25, P=0.007) in ADHD group compared to typically developing children in left and right occipital, respectively (Figure 3).

## 4. Discussion

This study aimed to compare the gamma band oscillations among patients with ADHD and healthy controls during facial emotion recognition. We expected to observe emotion recognition deficits in ADHD children. The results showed a significant (P<0.05) increased gamma band activity within occipital regions during the first time window (0–250 ms) compared to other sites. Also, we observed a significant (P<0.05) reduction in gamma band activity in ADHD children compared to normal group in response to facial expressions within occipital lobe. This study supported our hypothesis that individuals with ADHD were different from typically developing children during facial expression recognition, especially in early stage of facial emotion processing.

Consistent with our findings, several studies have shown a similar gamma-band increase in response to emotional pictures with occipital distribution (Keil et al., 2001; Keil et al., 2007; Garcia-Garcia et al., 2010). Balconi and Lucchiari (2008) as well as Aftanas, Varlamov, Pavlov, Makhnev and Reva (2002) reported a more emotional compared to non-emotional stimuli in posterior distribution of gamma oscillations. The results of the current study revealed that gamma band activity modulated by emotions compared to neutral, only in first time interval (0–250 ms) indicating early stage of facial emotion recognition but not late stage. Consistent with the current results about early gamma response (<250 ms), Martini et al., (2012) revealed the increase of gamma activity in the low gamma frequency band (30–45 Hz) for unpleasant picture compared to neutral visual stimuli at the shortest latencies (0–250 ms).

Also, Balconi and Pozoli (2009) showed an increased gamma band activity in response to emotions compared to neutral at 150–250 ms time interval with a peak at around 240 ms of latency. Keil et al., (2001) reported gamma band activity (30–45 Hz) at 80 ms poststimulus enhanced in response to unpleasant stimuli compared to neutral. These findings are consistent with our results that supported the role of early gamma response in assessing early stage of facial emotion processing. However, our findings did not reveal modulation of gamma band activity by emotion compared to neutral during the late stage. Unlike the current results, Martini et al., (2012) reported an increased gamma band activity (lower gamma frequency) in response to emotions (unpleasant compared to neutral) at the time interval of 250–500 ms. Also, they found two peaks of weak activation at high gamma frequencies (65–85 Hz) at 200 ms and 850 ms after stimulus onset. Keil et al., (2001) reported an increased gamma band activity (46–65 Hz) in response to unpleasant compared to neutral pictures at 500 ms. In fact, we did not analyze the 45–65 Hz or 65–85 Hz interval, that may be the reason for inconsistent results.

Also, the current results showed a significant diminished evoked gamma band responses only to anger and happiness emotions compared to neutral ones during early stage of processing (time window 0–250 ms) within occipital regions in ADHD group compared to healthy control ones. However, the current findings did not reveal any difference between groups in response to sad faces. Behavioral studies showed that ADHD children have deficits in emotional face recognition, especially negative (fear, anger, sadness) ones compared to healthy children (Singh et al., 1998; Cadesky et al., 2000; Corbett & Glidden, 2000, Dan & Raz, 2015). However, few behavioral studies did not report facial emotion recognition impairment (anger, happiness, sadness) in ADHD group compared to normal ones (Boakes, Chapman, Houghton, & West, 2007; Schwenck, et al., 2013).

Thus, the current results should be discussed with respect to early visual processing. Unfortunately, the relevant literature is scarce. Inconsistent with current results, William et al., (2008) reported adolescents with ADHD [(mean±SD) age: 13.79(2.33) years; range 8–17)] have been shown to display reduced occipital P1 component during all of expressions (fear, anger, sadness, disgust, happiness, or neutral) compared to normal group. The P1 component (in the ERP studies)is primarily involved in visual attention and initial sensory encoding (similar to role of early gamma responses according to Basar, 2012), localized in bilateral occipital areas and fusiform gyrus (Hillyard, Mangun, Woldorff, & Luck, 1995; Eimer & Holmes, 2002).

Few studies report reduced P100 amplitude in occipital regions in ADHD group (Barry et al., 2009; Nazari et al., 2010), probably indicating dysfunction of early visual pathways, which provide sensory input to the amygdala and may cause emotion recognition deficits. In the present study, participants’ Mean(SD) age was 9.21(1.13) years (age range was 7–11 years), which may be the reason for inconsistent results especially with William et al., (2008). In this regard, further research with other age groups or other gamma band frequencies (for happy and angry faces recognition) are recommended.

These findings show the important role of occipital gamma oscillations in facial emotion perception. The present study shows that children with ADHD have abnormality in brain function for early stage of emotional face recognition compared to normal children that can be caused by deficits in selective and sustained attention and early visual processing. These findings might provide preliminary evidence for future planning of interventional approaches for children with ADHD.
